# Testimonial to Prof. Dalton

**Published:** 1854-03

**Authors:** 


We find the following pleasant waif of information in the pages of the New York Medical Times. Dr. Dalton's friends in Buffalo, and elsewhere, will notice with pleasure the announcement of so flattering a testimonial, especially as they never would have learned it from the Dr. himself:

*Testimonial to Prof. Dalton.*—We are gratified to learn that the students at the Crosby Street Medical College have presented Prof. J. C. Dalton, Jr., with a silver box containing one hundred dollars in gold, as a proof of their high appreciation of the course of lectures on the "Physiology of Digestion and of the Nervous System," recently delivered by him in that institution, in conformity with the arrangement made with him for that purpose, as mentioned in our number for December. We regret that our limits prevent the insertion of the highly complimentary note with which the gift was accompanied, and the characteristic letter written in reply. The affair is highly creditable to both parties. Prof. D. is fast earning for himself a high reputation among us, both as a man of science and as a lecturer.

